# Hardiness in Family Caregivers During Caring From Persons With Alzheimer's Disease: A Deductive Content Analysis Study

**DOI:** 10.3389/fpsyt.2021.770717

**Published:** 2022-01-05

**Authors:** Lida Hosseini, Hamid Sharif Nia, Mansoureh Ashghali Farahani

**Affiliations:** ^1^School of Nursing and Midwifery, Iran University of Medical Sciences, Tehran, Iran; ^2^School of Nursing and Midwifery Amol, Mazandaran University of Medical Sciences, Sari, Iran; ^3^Nursing Care Research Center, Medical-Surgical Nursing Department, School of Nursing and Midwifery, Iran University of Medical Science, Tehran, Iran

**Keywords:** caregiving burden, family care, dementia, experiences, content analysis

## Abstract

**Objective:** This study was designed to describe the experiences of family Caregivers' hardiness in caring for Alzheimer's Patients.

**Methods:** The deductive content analysis method was performed between April 2020 and February 2021 in one of the teaching hospitals in Iran. Fourteen family caregivers of Alzheimer's patients were selected using purposive and snowballing sampling and the data were collected by semi-structured interviews. After that, data were analyzed using Elo and Kingas steps.

**Results:** The results of this study showed that based on the experiences of family caregivers, the family caregivers' hardiness in caring for Alzheimer's patients is a feature of cognitive ability to deal with stressful care situations and consists of five dimensions of commitment, control, challenge, communication and culture with 22 generic categories that they were nested into this five dimension.

**Conclusion:** Family caregivers' hardiness is a trait related to the individual and environmental factors, and the prevailing social and cultural conditions affect the individual's perception and experience of hardship and threats, as well as his/her understanding of protective factors and how to use them. Therefore, hardiness should not be interpreted as a simple approach regardless of culture.

## Introduction

In recent years, the world's older adult population has increased significantly due to increasing life expectancy and decreasing mortality and fertility rates. The World Health Organization (WHO) has reported that in 2019, 703 million people were 65 and older in the world, which is expected to double by 2030 to reach 1.5 billion people. However, this increase in the rate of the older adults population in developing countries such as Iran will occur faster than in developed countries, so that 79% of them will live in developing countries (1).

Aging is an inevitable stage in the life cycle of every individual, which is associated with a wide range of physiological, psychological changes, and especially decreasing in cognitive ability (2). The cause of reduced cognitive ability of the older adults can be due to reduced sensory abilities and mental related to age. Decreased sensory and intellectual abilities lead to the weakening of nervous processes in the older adults and make them prone to cognitive disorders (3). Alzheimer's Disease (AD) is one of the most common types of dementia, characterized by severe disorders of memory, thinking, and behavior (3). The prevalence of age-related Alzheimer's almost doubles every 5 years after age 65 (4).

Since the ability of persons with Alzheimer to perform personal activities decreases, they need to be supported by a caregiver who may or may not be a family member, a formal or informal care provider (5). Due to interdependence between family members, as well as declining household incomes, especially in developing countries such as Iran, and the lack of adequate supportive formal systems, family members assume responsibility for caring for these persons (5). Family caregivers are untrained individuals who do not receive any fee for providing services and at the same time meet the needs of the family and the needs of the care recipient (6). Giving that families provide more than 81% of the care needed by Alzheimer's patients and it is estimated that 7 out of 10 persons with Alzheimer's are cared for at home (2). Since the family caregivers accept another responsibility in addition to previous responsibility, they must be able to adapt to the situation, and this requires the caregiver's ability, competence, and responsibility, and can lead to acute and/or chronic illness in the long term (7).

Considering that the cultural and social conditions prevailing in each country affect care issues (8); In Iran, due to the Iranian culture that is associated with religion and encourages people to take care of their elders, the care of this group of persons are mostly performed by family caregivers (2). It should be noted that due to the lack of appropriate social structures and support systems to facilitate home care in Iran, these caregivers can have different experiences of care that studies have not considered (8).

### Background

Caring for Alzheimer's patients is an overwhelming task and a stressful situation for family caregivers; because caregivers play a key role in meeting the physical and emotional needs of these persons, and as the disease progresses, these tasks become heavier and lead to negative complications in the caregiver as a “burden” (5). The most common complications of caring burden in caregivers of Alzheimer's patients are depression, anxiety, stress, and burnout (9, 10). Moreover, negative side effects of the caring burden for the older adults with Alzheimer's include aggravation of symptoms such as irritability, aggression, hallucinations, delusions, and depression, which worsen the quality of life and increase mortality (10). Caring for Alzheimer's patients extremely burdened for family caregivers, some caregivers possess internal resources that modify the effects of caregiving stressors on psychological well-being and cope fairly well and even feel satisfied with the situation (7, 9). Hooker et al. suggested that personal characteristics should be the main factor in making such a difference in people's experience of care. Because it is the psychological characteristics that affect the meaning of care for each person (11).

Hardiness is one of the effective personality traits in stressful situations that have been considered by psychological theorists. This concept makes sense in the face of stressful situations and is considered as a moderator variable in the relationship between stress and the physical and psychological effects of that stressful situation (12). The concept of hardiness has been conceptualized as one of the main structures of personality to understand motivation, excitement, and behavior (12). Hardiness is a combination of attitudes and beliefs that motivate a person to do hard and strategic work in the face of stressful and difficult situations and facilitates turning adversity into an opportunity (13). Individuals with low hardiness are more likely to be affected by stressful situations, but individuals with high hardiness are less likely to experience adverse effects from the situation (14). Kobasa defined the hardiness model as consisting of three dimensions such as commitment (a sense of meaning in life), control (belief to able to influence the events of lives), and challenge (believing that change in life is natural and creates growth opportunities) (12).

Since the stressful situations that arise during the care of Alzheimer's patients are a unique and exhausting situation for family caregivers; hardiness is considered as an important personality trait to enable these caregivers to provide proper and appropriate care to these persons and to prevent problems for both the caregiver and the persons with Alzheimer (15); Because lack of hardiness not only leads to many physical and psychological problems such as fatigue, burnout, depression, sleep disorders, reduced quality of life, and even suicide for the caregiver, it can cause many problems for the Alzheimer's persons, including the possibility of neglect, abuse, poor quality care, ignoring vital needs, aggravation of the disease, and psychological and behavioral symptoms (16, 17). Therefore, knowledge of the status of family caregivers' hardiness in the care of persons with Alzheimer's disease and its effects on quality of life and patient care is essential. This study was designed to understand the experiences of family Caregivers' Hardiness in caring for persons with Alzheimer's disease in Iran based on the Kobasa hardiness model.

## Methods

### Design

This study was a deductive content analysis study to achieve to “explain the concept of family caregivers' hardiness in the caring of Alzheimer's patients” that was performed between April 2020 and February 2021.

### Participants and Setting

In total 14 family caregivers of Alzheimer's patients were selected by using purposive and snowballing sampling among the caregivers referring to the neurology clinic of hospitals of Tehran. All of the caregivers participating in the study were individuals who were responsible for caring of the patients with Alzheimer's disease at home. Nine participants were the daughter of a patient, two participants were the son of a patient and three participants were the spouse of a patient. The mean age of the participants was 54.57 years (see details in Table 1). The interview took place with the consent of the caregivers and in one of the clinic rooms or at their home.

**Table 1:** Demographic characteristics of patients and caregivers.

**Group**	**Variables**	**% (*n*)**
Caregiver demograohic characteristics	**Age**	54.57 ± 14.64
	**Sex**	
	Female	71.4% (10)
	Male	28.6% (4)
	**Marital status**	
	Single	21.4 % (3)
	Married	71.4 (10)
	Divorced	0% (0)
	Widowed	7.1% (1)
	**Relationship**	
	daughter	64.3 % (9)
	Son	14.3 % (2)
	Wife/midwife	21.4 % (3)
	**Lifestyle**	
	Independent	35.7 % (5)
	With patient	64.3 % (9)
	**Education**	
	Illiterate	14.3% (2)
	Less than diploma	0% (0)
	Diploma	28.6 % (4)
	BS/MS/PhD	57.1 % (8)
	**Occupation**	
	Unemployed	14.3 % (2)
	Employed	37.5 % (5)
	Retired	21.4 % (3)
	Housewife	21.4 % (3)
	Volunteer	7.1% (1)
	**Duration Of Caregiving (hour per day)**	9.78 ± 3.04
Patients demograohic characteristics	**Age**	79.07 ± 6.7
	**Sex**	
	Female	64.3 % (9)
	Male	35.7 % (5)
	**Duration Of Illness**	6.07 ± 3.66
	**Number Of Children**	5.07 ± 2.01

### Data Collection

In this study, the data collection method was Face-to-face or via Skype or WhatsApp, in-depth and semi-structured interviews conducted by the first author using a combination of targeted (main categories of hardiness model) questions and open-ended questions. Interview duration was 35–90 min (see the list of questions in Table 2). As the interview progresses, for more details and deepening interviews, we used to explore questions such as: “can you explain more about this?”, “can you give an example?”, “when you say… what do you mean?” “are there any other things you want to talk to me about?”. Participants were interviewed until data saturation was reached. It has reached a degree of saturation whenever no new categories or suitable theme emerges, or in other words, when the researcher deeply discovers each category and determines its different characteristics and dimensions in different situations (18).

**Table 2:** List of pre-interview open-ended and targeted questions.

**Open-ended questions**	**Targeted questions**
Please describe a day from morning to a night spent caring for your patient?	What makes you committed to caring for your patient? (main category of Commitment.)
What makes you take care of your patient?	How do you control the stressful situation of caring for your patient? (main category of control)
What do you do to deal with care-related problems?	How do you look at patient care? (main category of the challenge)

### Data Analysis

Data were analyzed based on the proposed Elo and Kyngäs in three phases (19).

*Preparation phase*: This phase consists of two parts: selecting the unit of analysis and finding the logical connection of the data with the whole subject. The transcript interviews were considered as a unit of analysis. So, immediately after the end of each interview, the interview was transcribed word by word on paper. Then, transcribed texts were reviewed and re-read several times to immerse in the data to better understand what was going on in the data and understand the participants' feelings and experiences by asking frequently asked questions e.g., what is happening? who speaks? where is this happening? when did this happen? what happened? and why? A general sense of the text was obtained and then the analysis was performed using a deductive approach.

*Organization phase:* In this phase, the researcher developed an unconstrained matrix derived from the concepts of the Kobasa hardiness model. At this phase, after explicit and implicit concepts were identified in form of sentences or paragraphs from the participants' statements, the researcher assigns preliminary codes to associated meaning units. The next process after coding was to integrate the same preliminary codes into more comprehensive classes. Continuous comparison in the analysis process helped to integrate and summarize similar codes in the form of primary categories and by continuing, continuous comparison of primary categories based on similarities; differences, and proportions, abstraction was done and categorized into generic categories. Generic categories nested into the main categories in the matrix or new categories were created. These processes of coding, categorizing, and extracting themes were performed by the contribution of all three authors (L.H, M.A.F & H.S.N) independently. The final themes and subthemes were extracted by regular meetings discussions. MAXQDA software was used to manage data and the facility of the process.

*Reporting phase:* All stages of deductive content analysis and the findings obtained in the present study, including the sampling process, participants' characteristics, data collection, data analysis were reported.

### Trustworthiness of Stud

The quality of data and findings were evaluated by credibility, dependability, confirmability, and transferability (20). For ensuring and evaluating the rigor of the study, following approaches were used: an 11-month engagement period in the research setting, member checking, peer debriefing, recording step by step, the interviews, transcribing them immediately after each interview, and evaluating the process of analysis by qualitative research experts.

## Results

In this study some generic categories were nested into the main categories of commitment, control, and challenge; but we developed two new main categories such as connection and culture. In the main category of commitment, the “emotional tendencies,” “external motivation,” “intrinsic motivation,” “understanding care,” and “responsibility” were developed. In the main category of control, “knowledge of the disease,” “promoting knowledge of the caring,” “protecting the patient's body and mind,” “self-management,” “condition management,” “caring ability,” and “effective communication with the patient” was developed. In the main category of challenge, the “high values of caring,” “gaining skills of caring,” and “mental well-being” were developed. In the new main category of connection, the “supportive family,” “supportive people,” and “family supporter” were developed. Finally, in the new main category of culture, “individual values,” “adherence to ethics virtues,” “religion,” and “social values” were developed (see details in Table 3).

**Table 3:** Dimensions of hardiness concepts in family caregivers.

**Main categories**	**Generic categories**	**Initial categories**
Commitment	emotional tendencies External motivation Intrinsic motivation Understanding care responsibility	attachment to family attachment to patient sense of belonging Motivate the family Motivate patient's doctor Motivate the others Motivate the quality of care Motivate the religious beliefs Gain motivation from the pattern of past relationships Perceived care pressure Perceived financial pressure from care Endurance in a caring role Effective caregiver role Accepting the position Acceptance of duty
Control	knowledge of the disease promoting knowledge of the caring Protecting the patient's body and mind self-management Condition managemen t Caring ability Effective communication with the patient	Awareness of the nature of the disease Awareness of the client's cognitive problems Awareness of the client's physical problems Awareness of the client's psychological problems Awareness of the progression of the disease Awareness of care tips Seek awareness of non-human resource care Gain awareness of human resource care Share information with the family Maintaining the patient's mental health Maintain the patient's cognitive power Maintaining the patient's physical health Trying to keep the patient active Calming the mind Keep calm through distance-term care Keep calm through interaction Performance control Rational approach to the situation Provide appropriate care for the patient Problem solving skills Balance life with a caring role Functional capability Cognitive ability Psychological empowerment Involve the patient in the affairs Positive communication with the patient
Challenge	High values of caring gaining skills of caring Mental well-being	Gratitude Admonitions to children Reward faith Spiritual excellence Self-care Acquire emotional support skills Acquire functional skills Experience pleasant emotions Satisfaction with the caring role
Connection	supportive family supportive people family supporter	Family financial cooperation Family cooperation in tasks Effective family interaction Consult a specialist Get help from others Family support in care Family psychological support
Culture	Individual values Adherence to ethics virtues Religion social values	Being conscientious Being grateful Understanding the patient's self-sacrifice Preserving the dignity of the client Ethical considerations in interacting with the client Religious beliefs affecting care Religious Behaviors Influence of family norms Learning from life

### Commitment

Commitment in family caregivers refers to a person's ability to engage with a situation instead of relinquishing it based on his/her emotional tendencies, being influenced by internal and external motivations, understanding care, and being responsible.

#### Emotional Tendencies

Based on the experience of family caregivers, what caused them to become involved in the caring situation and not give up caring for their patient was their attachment to family, patient, and sense of belonging, including love to the patient. They showed attachment to their family by loving them, understanding the situation of other family members, and trying to reduce the burden of caring for them and fulfilling their responsibilities to the family. These caregivers showed attachment and a sense of belonging to their patient by living with and accompanying the patient as much as possible, quitting their job to care for the patient, devoting their time of rest to persons, and loving and depending on the patient.

“*What makes me take care of my mother is love, I go to my mother and with love, and that is, I fall in love when I see my mother”. (Participant 4)*

#### External Motivation

Motivations gained from family, the patient's physician, and others around them such as relatives and friends made these caregivers more committed to caring for their persons. The family of these caregivers motivates them to continue caring by trusting the caregivers in care and consulting with them in all matters related to the patient, understanding the burden of the caregiver and thanking the caregiver, as well as comforting the caregiver in times of fatigue and distress. Friends and relatives also motivate caregivers by reflecting the quality of caring to caregivers and appreciating from caregivers. It is noteworthy that almost all caregivers emphasized the importance of motivating the patient's physician to care for their ability to continue care. This motivation was created through physician feedback and satisfaction about the quality of care provided by caregivers and provided caregivers with more motivation and ability to care.

“*Whatever I say, they accept and trust me and I say this is good for the mother, they accept. If my mother gets worse, they will never complain to me that you made it. Well, that motivates me.” (Participant 2)*

“*When I take him doctor, he gives me a lot of hope, he does not let me be disappointed, he says you worked very well with him, he tells me that considering that he has these conditions, his process is very good, it increases my motivation, it increases my energy, I try harder”. (Participant 7)*

#### Intrinsic Motivation

Perceived quality of caring such as improving the patient's condition, the patient's condition is better compared to other persons, persuasive religious beliefs such as have a religious reward, and patterns of past relationships were the intrinsic motivation that causes family caregivers to continue to care.

“*When I see that what I have done, has made her condition better, i see that she used to be depressed, now she is better, it makes me more motivated to try harder.” (Participant 12)*

“*I think they might consider me a religious reward, and that motivates me”. (Participant 1)*

#### Understanding Care

According to family caregivers, what enables them to properly cope with the difficulties of care and continuing their caring successfully is to understand the care needed and its challenges including the pressures of care and its financial pressures.

“*I knew about my mother that this would be a difficult situation, it would be easy at first, but it would get harder and harder over time, so I was prepared”. (Participant 10)*

#### Responsibility

The experience of these family caregivers demonstrated that they showed their responsibility to care for their patient by having endurance in the caring role, effectively taking on the caring role, accepting the position, accepting their duty, and these combined factors indicate that they are committed to the position of career.

“*I mostly think that what makes me take care of my father now is that I think it is a moral, human and filial duty. In fact, I take care of my father because of its moral, human and filial duties”. (Participant 1)*

“*I fully understood these conditions and accepted that they exist”. (Participant 4)*

### Control

Control refers to the caregiver's ability to influence the situation based on knowledge of the disease, promoting knowledge of the caring, protecting the patient's body and mind, self-management, condition management, caring ability, effective communication with the patient.

#### Knowledge of the Disease

From the caregivers' point of view, being aware of the nature of the disease, cognitive and psychological problems, the course of the disease, and the points of care required were important in the ability of individuals to control the situation and affect it effectively.

“*Well, I'm aware of my mother's illness, I know that Alzheimer's disease is losing its ability, and this disease causes a series of disabilities in its movement”. (Participant 2)*

#### Promoting Knowledge of the Caring Role

Searching for information from non-human resources such as (the internet, books, articles), and human resources including (attending caregiver training classes, attending seminars and workshops), and sharing information with family members and other caregivers were some of the ways caregivers improve knowledge to increase their ability to control the situation.

“*I tried very hard to get information about the disease from the internet, I bought a book about the disease and read it so that I could control it”. (Participant 8)*

#### Protecting the Patient's Body and Mind

During care, family caregivers stated that by trying to improve and maintain the patient's mental and physical health, as well as helping to improve his/her cognitive status by reminding him/her of information and trying to keep him/her active, they tried to improve the situation and illness and prevent from the situation getting worse.

“*That is, we constantly tried to maintain his health by encouraging father and asking him to do a series of things”. (Participant 1)*

#### Self-Management

During care, family caregivers tried to maintain their composure by doing favorite activities such as yoga, having leisure time, socializing with relatives when they were tired, and controlling the performance of their care and in this way take control of the situation.

“*I have a series of courses such as health courses, meditation courses, TM, breathing meditations that I try to relax with. In fact, each of these helped me to be calm”. (Participant 6)*

#### Condition Management

Family caregivers stated that they tried to manage the situation through a rational approach to the situation, providing appropriate care to the patient's condition, using different problem-solving techniques, and balancing personal life with the caring role.

“*With the management, we tried to solve the problems of forgetting the father. For example, every day that my father wanted to go to work, we checked that is his mobile phone charged? does the workplace key come with it?” (Participant 1)*

#### Caring Ability

Family caregivers stated that by trying to improve their capabilities in functional, cognitive, and psychological dimensions, they acquired the necessary skills and abilities to control the situation and provide appropriate care.

“*We have to be very patient, we have to be very resilient, we have to know that this responsibility that we have taken on is hard work and we have to accept it, we have to move forward and not be afraid. We must be strong-willed, not quick to get angry, and quick to get tired. It requires patience and endurance so that one does not get tired soon”. (Participant 3)*

#### Effective Communication With the Patient

Family caregivers stated that during the care period, they tried to control the situation and have a positive impact on it by establishing a positive relationship with the patient and involving him/her in doing things.

“*I always try to be very supportive, that is, I try to love him very much, so I try to treat him by being kind, by hugging, by using kind words to talk to him”. (Participant 3)*

### Challenge

Challenge refers to the caregiver's ability to turn the stressful situation into an opportunity to grow for them based on an acquisition of the high values of caring, gaining skills of caring, mental well-being.

#### High Values of Caring

Family caregivers see the caring situation as an opportunity for gratitude and compensation for the patient's past troubles and to teach vital traits such as patience, morals, and understand the importance of respecting adults to their children, as well as an opportunity to improve their spirituality including moving away from worldly issues and making positive changes to their way of life.

“*Parents work very hard for us children, maybe my mother, if she was a woman who only cared for herself, would not care about her children, maybe she would not get Alzheimer's, these parents work for me, in hardships and joys and everything, they were with me, so now is the time to compensate them”. (Participant 2)*

“*I see this as a good opportunity for myself; an opportunity that I am very calm when I am with her and I am free from all worldly issues and works”. (Participant 7)*

“*When my father contracted this disease, I have changed my way of life in my work, in my life, and I believe that it was right”. (Participant 8)*

#### Gaining Skills of Caring

Family caregivers stated that during their patient care, they acquired many skills including how to take care of themselves to prevent future illness, how to deal with a patient, and how to care for a patient.

“*I gained a lot of experience. Maybe now I know things that I did not think, I would want to do or learn in the past. Now, for example, I have learned the type of caring from a patient, feeding the mother, and all the abilities that a nurse needs, experimentally. I learned a lot of skills”. (Participant 9)*

#### Mental Well-Being

Mental well-being is an important structure related to the interpretation of personality and is defined as a positive assessment of life and the balance between positive and negative emotions. Family caregivers achieved this mental well-being during care by gaining satisfaction from their caring role and experiencing pleasant emotions such as the experience of love during caring, not having a guilty conscience, and the best opportunity to see care for themselves.

“*Just I feel that I am taking good care of my patient now and I am trying to do the responsibility that I have well, it makes me feel good”. (Participant 2)*

“*I do not regret it at all, I am not upset at all that I agreed to take care of them... I am happy to take care of them, I do not have a guilty conscience that I did not do everything I could for them”. (Participant 11)*

### Connection

The connection refers to the acquisition of power and the ability to influence the stressful position of care in family caregivers through the existence of a supportive family, supportive people, and family supporters.

#### Supportive Family

Family caregivers tried to gain the strength and power to continue caring by communicating with family members and engaging them in caring and receiving financial assistance.

“*Well, my family is with me, when I am tired, my sister comes and does this with her and I rest a little and come back with more strength”. (Participant 2)*

#### Supportive People

Family caregivers also tried to increase their strength and ability to cope with the difficulties of the situation by consulting specialists in times of trouble and receiving help from others.

“*Well, I hired a nurse, I wanted her to help me and reduce my burden of care, and I have more control over the condition, and not to mess up the house”. (Participant 12)*

#### Family Supporter

The caregivers stated that they also provided support to their family members during their care and supported them psychologically and physically. They supported them to maintain the morale of their family members and relieve their feelings of remorse, as well as to reduce their burden of care.

“*I pretend to my mother, family, siblings that everything is going well and my father will be fine so that they do not lose hope, but I know this is not the case”. (Participant 8)*

### Culture

Culture also includes a set of individual values, adherence to ethics virtues, religion, social values of the caregiver that affect his/her ability to cope with the stressful situation during care.

#### Individual Values

Individual values such as conscientiousness, gratitude, and understanding of the patient's self-sacrifice were among the characteristics that enhanced the ability of caregivers to cope with stressful situations.

“*I think everyone has a duty and they have to do their duty properly and I have always tried to do my duty properly, I always think that I am a daughter and I must do this. It is my duty to my father and I have to take care of him now”. (Participant 1)*

#### Adherence to Ethics Virtues

Caregivers stated that adherence to ethics, including maintaining the patient's dignity and maintaining ethical considerations in interacting with the patient is an important factor that can affect the ability of individuals to cope effectively with stressful situations.

“*It is important for me and I try to treat my mother like a mother and child and the respect and those values and those credentials and trusts remain and my mother's respect and dignity is not lost”. (Participant 2)*

#### Religion

Religious beliefs and behaviors were other factors that helped caregivers to moderate the severity of the stressful situation for them and increase their ability to cope effectively with the stresses created during care.

“*I believe that god has never helpless me, I know that god has always been by my side and helps me and has given me this strength and ability”. (Participant 5)*

#### Social Values

Family norms learned by caregivers throughout their lives, abilities learned in life's hardships, and norms governing the community in which they live including respect for the older adults and helping the disabled were social values that helped ability caregivers to get hardier during care.

“*We have always learned that we must be behind each other everywhere and always help each other. We vowed to always be together. Maybe that helped us”. (Participant 13)*

## Discussion

This study was the first study to explain the experience of family caregivers' hardiness in caring for patients with Alzheimer's in Iran. The results of the present study confirmed three dimensions of the Kobasa of hardiness model (12), but the notable point is that our finding explained the hardiness concept in family caregivers has two additional dimensions including connection and culture. The dimension of connection (21); could be an important and influential dimension on individuals' hardiness in dealing with stressful situations. Individuals gain part of their power and ability to deal with stressful situations as a result of connection with other members of society, therefore, the connection is one of the factors that seem to be able to play an important role in creating and maintaining hardiness. In this study, family caregivers stated that during their patient care period, by seeking and receiving help from family members, friends, relatives, and health professionals including nurses tried to cope effectively with the stress of the situation and reduce the burden and its effects on themselves and can become hardier in dealing with the situation. Clark supports this finding; so that they asserted hardy caregivers used more overall transformational coping and help-seeking and receiving more assistance from their family. Also, they found that the family interaction in handling stressful events has a positive effect on the coping strategies of the family caregiver (16). Furthermore informal support networks including family support reduce negative caregiver health outcomes (22). This finding is also in line with the results of another study that confirmed caregivers try to cope with the challenges of the situation in terms of going to support groups and utilizing other community resources (17).

Some researchers have also observed significant differences in stress levels and coping mechanisms used by individuals in different cultures (23, 24). Hardiness is a characteristic related to the individual and his/her environment because the social and cultural conditions governing the individual affect his/her perception and experience of hardship and threat, as well as his/her understanding of protective factors and how to use them and it can mean hardiness to him/her (23). The present study also confirmed this dimension as influential in shaping the hardiness of family caregivers in caring for Alzheimer's patients. Family caregivers in this study emphasized that their values including (conscientiousness, gratitude, and understanding of the patient's self-sacrifice), their religious behaviors and beliefs, and social values including family norms greatly influence their hardiness in dealing effectively with care stress and increase their ability to deal appropriately with the situation and reduce the negative effects of the care burden. Religion provides conditions that are useful for improving the health and well-being of people in stressful situations. Previous studies also have been shown, religion makes a person more hardy in the face of problems and has more control over his/her actions and behavior (25, 26). In general, the unique lifestyle of religious people empowers them to evaluate events as less stressful, or after stress, to see it as an opportunity to grow and strengthen their spirit (26).

Commitment is the first dimension of hardiness in the Kobasa model and refers to a tendency to engage in life activities and have a real interest in curiosity about the world around (27). Family caregivers showed commitment to caring for your patient through emotional attitudes including attachment to the patient and family and a sense of belonging to the patient, motivation from external factors including family, physician, and others, motivation from internal stimuli including quality of care perceived by self, religious beliefs and patterns of past relationships with the patient, understanding of care and responsibility by accepting duty, accepting position, perseverance in the role and effective leadership. Researchers had shown commitment is a unique and powerful predictor of psychological distress; Because caregivers' desire to feel a sense of belonging to caring activities and a sense of belonging to their condition and persons, leads to stability and acceptance of the negative aspects of care by them (15).

Control is the second dimension of hardiness in the Kobasa model. It is as an inner desire to believe and act that one can influence the events of one's life, and this belief in influence occurs in one's effort (13). Family caregivers demonstrate their ability to control the situation by having knowledge of the physical, cognitive, psychological, and care tips needed, improving the knowledge and caring from human resources such as specialists and non-human resources such as books and articles, trying to protect the body and mind the patients, managing mind and performance themselves, managing the situation by using appropriate problem-solving strategies and tailored to the situation, and finally improving their functional, cognitive and psychological abilities and gaining the caring ability. Caregivers' knowledge, self-confidence for managing chronic and acute health conditions of care recipients, alerting clinicians about worrisome changes, self-management, and practicing self-care skills have a significant effect on controlling the situation (28).

Challenge is the third dimension of hardiness in the kobasa model and refers to beliefs that change, rather than being stable, as a natural state of life, creates opportunities for personal growth rather than a threat to one's security (27). Family caregivers confirmed this dimension. They stated that this period of patient care provided them with an opportunity to make up for the patient's past troubles, gain religious rewards through this situation, be able to teach their children good morals and respect for the elders, and let their spirituality grow, as we put them in a category called the high values of care. Family caregivers also stated that this period of care provided them with many skills in how to properly and effectively communicate with an Alzheimer's patient and how to provide care for them, so they stated that a self-taught nurse was now trained. Ultimately, this period of care provided them with an opportunity for mental well-being through the experience of pleasant emotions and the creation of a sense of satisfaction with the role of care and life and the absence of remorse. The previous study confirmed our finding that caregivers who viewed their caregiving activities as an important, meaningful, and rewarding situation for them, were less distressed (15).

One another interesting point in this study was that the participants stated that the existence of community support such as the support of formal community organizations, the support of the health organizations, and social groups can help significantly in improving their hardiness, enhancing their ability to care for patients with Alzheimer's disease, coping well with the care situation, and reducing the negative effects of the care. Based on participants' statements as well as findings from previous studies (29), this social support did not exist in our society. Therefore, we suggest that the impact of this factor on hardiness be further investigated in communities that have organizations that support family caregivers, especially caregivers of Alzheimer's patients. Furthermore, this study includes qualitative information and cross-cultural analysis of the results is not carried out. Therefore, we suggest it considers in future study.

## Relevance to Clinical Practice

Since patients with Alzheimer's disease are mostly cared by family members, nursing care programs should focus on improving the adaptability and hardiness of these caregivers. Recent studies in the nursing profession suggest that the 10 creative factors of Watson's theory in nursing including the formation of the humanistic-altruistic system of values, nurturing faith and hope, cultivation of sensitivity to one's self and others, development of a helping-trusting human relationship, promotion, and acceptance of the expression of positive and negative feeling, use of the creative problem-solving caring process, promotion of transpersonal teaching-learning, provision of supportive, protective, or corrective mental, physical, sociocultural, and spiritual, assistance with the gratification of human needs, and allowance for existential- phenomenological-spiritual forces (30) are related to the categories obtained in this study as a result of examining the experiences of caregivers. So nurses and therapists can plan to improve the family caregiver's hardiness with forming future caregiver hardiness research by considering categories obtained in this study and using Watson's theory as a guide.

## Conclusion

Concept of hardiness in Iranian family caregivers had three dimensions expressed by Kobasa. But the remarkable point was the impact of the important role of culture in the formation of this feature in family caregivers in Iran. Hardiness by being influenced by religion and personality formed in Iranian culture, allows people to use attitudes and behavioral and emotional values to develop many individual characteristics such as empathy, understanding, responsibility, flexibility, attention, cooperation, tolerance, intelligence efficiency, and optimism and be able to properly deal with stressful situations and be safe from depression and stress. Also to improve the hardiness of family caregivers and reduce the negative effects of the care burden in the caring of Alzheimer's patients, nurses should use their emotional tendencies, helping them by strengthening their social networks, and finally taking into account their values, beliefs, and cultural norms.

## Data Availability Statement

The original contributions presented in the study are included in the article/supplementary material, further inquiries can be directed to the corresponding author/s.

## Ethics Statement

The studies involving human participants were reviewed and approved by the Ethics Committee of Iran University of Medical Sciences (IR.IUMS.REC.1398.1229). The patients/participants provided their written informed consent to participate in this study.

## Author Contributions

All authors listed have made a substantial, direct, and intellectual contribution to the work and approved it for publication.

## Conflict of Interest

The authors declare that the research was conducted in the absence of any commercial or financial relationships that could be construed as a potential conflict of interest.

## Publisher's Note

All claims expressed in this article are solely those of the authors and do not necessarily represent those of their affiliated organizations, or those of the publisher, the editors and the reviewers. Any product that may be evaluated in this article, or claim that may be made by its manufacturer, is not guaranteed or endorsed by the publisher.
